# Effect of the TAAR1 Partial Agonist Ralmitaront on Presynaptic Dopamine Synthesis Capacity Measured Using [^18^F]DOPA PET in Naïve and Cocaine-Treated Mice

**DOI:** 10.1177/15353508241299546

**Published:** 2024-12-18

**Authors:** David R. Bonsall, Michelle Kokkinou, Els F. Halff, Grazia Rutigliano, Sac-Pham Tang, Mattia Veronese, Elaine E. Irvine, Dominic J. Withers, Lisa A. Wells, Sridhar Natesan, Irene Gerlach, Štefan Holiga, Marius C. Hoener, Oliver D. Howes

**Affiliations:** 1 Psychiatric Imaging Group, MRC Laboratory of Medical Sciences (LMS), London, UK; 2 Invicro, Burlington Danes, Hammersmith Hospital, London, UK; 3 Institute of Clinical Sciences, Faculty of Medicine, 4615 Imperial College London, London, UK; 4 Department of Psychosis Studies, Institute of Psychiatry, Psychology and Neuroscience, King’s College London, London, UK; 5 Department of Neuroimaging, Institute of Psychiatry, Psychology and Neuroscience, King's College London, London, UK; 6 Department of Information Engineering, 9308 University of Padua, Padua, Italy; 7 Metabolic Signalling Group, MRC Laboratory of Medical Sciences (LMS), London, UK; 8 Roche Pharma Research and Early Development, Roche Innovation Center Basel, F. Hoffmann-La Roche Ltd, Grenzacherstrasse, Basel, Switzerland; 9 South London and Maudsley NHS Foundation Trust, Camberwell, London, UK; 10 H. Lundbeck A/s, St Albans, UK

**Keywords:** schizophrenia, dopamine, cocaine, TAAR1 agonist, PET imaging

## Abstract

**Purpose:**

Elevated dopamine synthesis capacity is part of the pathophysiology of schizophrenia thought to underlie psychosis. Drugs that reduce this phenomenon could thus be potential treatments for these disorders. In this study, we evaluated the ability of the trace amine-associated receptor 1 (TAAR1) partial agonist ralmitaront to reduce presynaptic dopamine synthesis capacity.

**Procedures:**

Ralmitaront (3 mg/kg, i.p.), a TAAR1 partial agonist, was evaluated using [^18^F]DOPA PET for its ability to modulate presynaptic dopamine synthesis capacity in naïve mice as well as mice in an induced hyperdopaminergic state following acute cocaine administration (20 mg/kg, i.p.).

**Results:**

Cocaine treatment on its own did not induce elevated dopamine synthesis capacity when compared to the control group. Pretreatment with ralmitaront significantly reduced dopamine synthesis capacity when given either alone (44%) or in combination with the psychostimulant cocaine (50%) when compared to the control group.

**Conclusions:**

The TAAR1 agonist ralmitaront reduces striatal dopamine synthesis capacity, indexed as Ki^Mod^, both in naïve animals and when given prior to acute cocaine. This indicates the potential of TAAR1 agonism to address disorders characterized by striatal hyperdopaminergia.

## Introduction

Current treatments for schizophrenia and related psychotic disorders are all dopamine D_2/3_ receptor blockers.^1^ They are ineffective for some patients and associated with significant side effects in many others, highlighting the need to develop nondopamine D_2/3_ blocking alternative treatments.^2,3^ Elevation of striatal dopamine synthesis capacity (DSC) has been linked to positive symptomatology and antipsychotic response in schizophrenia.^4–6^ This indicates that reducing DSC is a therapeutic target for developing novel approaches to treat psychotic disorders.^6,7^

Psychostimulants like amphetamine and cocaine are known to increase extracellular dopamine predominantly by blocking dopamine transporter's (DAT) actions and hence they have been widely used as a screening model for antipsychotic agents in rodents.^8^ TAAR1 (trace amine-associated receptor 1) is expressed in the mesolimbic system, especially in dopamine neurons, and its activation has been shown to reduce dopamine neuron activity.^9^ Moreover, selective TAAR1 agonists are known to decrease cocaine-induced locomotor activity in rodents.^10^ This led to our hypothesis that hyperdopaminergia could be decreased by TAAR1 agonism possibly by decreasing DSC.^11,12^ In this context, we used 3,4-dihydroxy-6-[^18^F]fluoro-l-phenylalanine ([^18^F]-DOPA) PET imaging to investigate the effect of TAAR1 partial agonist, ralmitaront, on DSC in naïve animals and in animals that have received cocaine.^13^

## Material and Methods

### Experimental Animals and Ethics Statement

Male C57Bl/6J mice aged 9 to 10 weeks and obtained from Charles River, Kent, UK, were group-housed under 12:12 hours light: dark cycle. Food and water were provided ad libitum and room temperature was maintained between 20 °C and 24 °C and relative humidity was kept between 45% and 65% with adequate nesting and environmental enrichment material provided within cages. Experimental procedures were performed in accordance with the UK Animals (Scientific Procedures) Act 1986 after institutional animal welfare ethics and UK home office approval (Project license PE0206466).

### Micro PET Imaging

**Figure 1.**
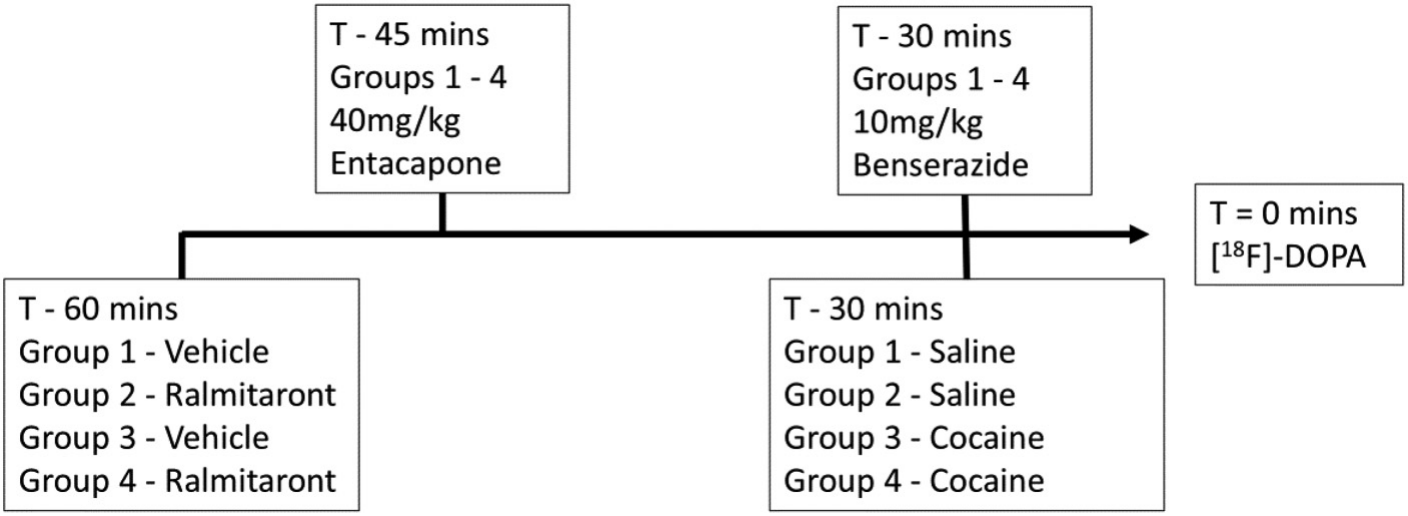
Treatment groups and timeline of drug administration prior to positron emission tomography (PET) scanning. Group 1 serves as the control group.

Mice were randomly assigned to one of 4 groups as depicted in Table 1. Prior to the PET scan, mice underwent jugular vein cannulation for radiotracer delivery and received a total of 4 intraperitoneal injections 30 to 60 min before [^18^F]-DOPA administration (Figure 1). These included the TAAR1 agonist (3 mg/kg ralmitaront, also known as RO6889450 and RG7906, Roche Ltd, CAS number 2133417-13-5) or vehicle (0.3% Tween80), peripheral DOPA metabolism inhibitors (entacapone 40 mg/kg and benserazide 10 mg/kg), and cocaine (20 mg/kg) or saline vehicle. Dynamic PET scans of mice were started concurrently with the delivery of 1-10MBq [^18^F]-DOPA, given intravenously, and acquisition lasted for 120 min.^14^ All laboratory chemicals were purchased from Sigma Aldrich, UK, and [^18^F]-DOPA was produced in-house at Invicro.

**Table 1.** Treatment Groups.

	Vehicle + saline (control group) (n = 10)	Ralmitaront + saline (n = 10)	Vehicle + cocaine (n = 10)	Ralmitaront + cocaine (n = 11)
Weight (g)	24.7 ± 0.8	24 ± 0.6	26.1 ± 0.8	24.9 ± 0.6
Injected dose (MBq)	4.3 ± 0.8	4.2 ± 0.7	4.7 ± 0.9	4.4 ± 0.6
Dose/g (MBq/g)	0.174 ± 0.03	0.175 ± 0.03	0.181 ± 0.03	0.177 ± 0.03

The values mentioned are Mean + SEM. A one-way ANOVA indicated no significant differences (*p* > .5) in animal weight or injected dose across the 4 treatment groups. Abbreviations: SEM, standard error of the mean; ANOVA, analysis of variance.

### PET Data Analysis

Data obtained from the dynamic PET scan were histogrammed and reconstructed using filtered back projection. Attenuation correction of PET signal was carried out using the computer tomography (CT) acquisition for each subject. The percentage-injected dose was corrected for body weight and activity injected for each mouse to provide standardized uptake values (SUV). Using the CT-aligned reconstructed PET image, regions of interest (ROI) were manually drawn on the left and right striatum and the cerebellum (used as a reference region) by the analyst blind to the condition. Time activity curves of the [^18^F]-tracer activity within the ROIs were generated. Dopamine synthesis capacity (indexed here by a modified version of net tracer uptake, Ki^Mod^)^14^ was determined using extended Patlak graphical analyses of the irreversible component of the time activity curve (calculated from 20 to 90 min of the PET scan), comprising the accumulation and conversion of [^18^F]-DOPA to [^18^F]-dopamine which takes into account an estimation of *k*_loss_, the rate at which signal is removed from the system due to metabolism of [^18^F]-dopamine.^15^ A representative PET scan, time activity curve, and Patlak plot of a mouse from Group 1 is available in Supplemental Material 1.

### Statistical Analysis

Group mean and their standard error of mean (SEM) have been reported in the results. Differences between group variables were tested using one-way analysis of variance (ANOVA) followed by Tukey's post hoc test if needed. SigmaPlot software (Version 14.5) was used for the statistical analysis.

## Results

**Figure 2.**
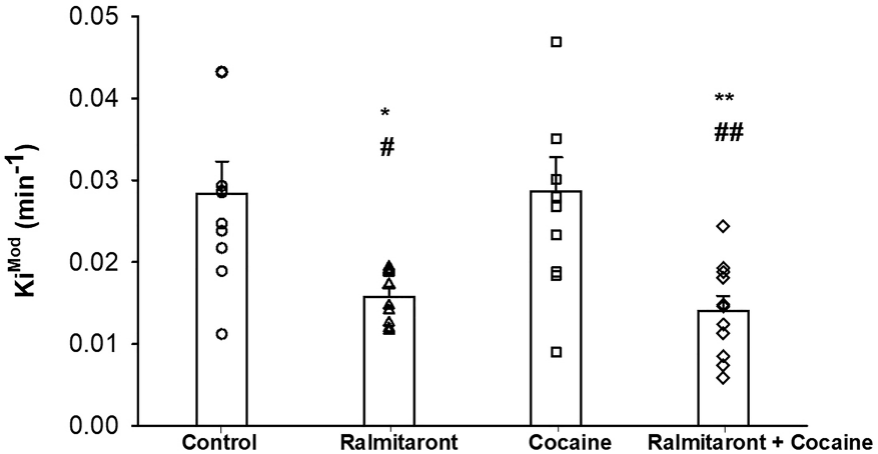
Effect of TAAR1 agonist ralmitaront, cocaine, and their combined effect on dopamine synthesis capacity (Ki^Mod^).

There were no significant differences in animal weight or injected dose across the 4 treatment groups (Table 1). One-way analysis of variance (ANOVA) followed by Tukey's post hoc test of Ki^Mod^ values among groups is depicted in Figure 2. The ANOVA showed a significant difference among the groups (*p* < .001). Ralmitaront was given alone (Group 1 vs Group 2) significantly reduced Ki^Mod^ by 44% (Control Ki^Mod ^= 0.028 ± 0.004 min^−1^, ralmitaront = 0.016 ± 0.001 min^−1^, *p* < .05). DSC in the cocaine-treated group was not statistically different from controls (Group 1 vs Group 3, 0.029 ± 0.004 min^−1^). However, pretreatment with ralmitaront followed by cocaine resulted in a 50% reduction in Ki^Mod^ (Group 1 vs Group 4 0.014 ± 0.002 min^−1^) from control (*p* < .01). ANOVA of *k*_loss_ among the groups showed significant difference (*p* < .05; Group 1 = 0.0221 ± 0.00440, Group 2 = 0.0155 ± 0.00145, Group 3 = 0.0289 ± 0.00394, and Group 4 = 0.0128 ± 0.00271). Post hoc revealed a significant difference (*p* < .01) only between Group 3 (cocaine treatment) versus Group 2 (ralmitaront treatment) and (*p* < .05) between Group 3 (cocaine treatment) and Group 4 (pretreatment with ralmitaront + cocaine).

Data shown as mean ± SEM. ^* ^indicates differences from control, ^# ^indicates differences from cocaine, ^*/#^*p* < .05, ^**/##^*p* < .01. Abbreviations: TAAR1, trace amine-associated receptor 1; SEM, standard error of the mean.

## Discussion

The results of the present study indicate that ralmitaront reduces DSC in mice both under control and cocaine-stimulated conditions. The fact that ralmitaront is able to reduce DSC in mice extends our prior finding that activating TAAR1 can be used to normalize elevated DSC.^16^ There is some inconsistency in the findings on the effects of dopamine D_2/3_ blockers that are currently used for treating psychosis on DSC, with one study showing a reduction in some parameters,^17^ but the majority of studies show no significant effects of a range of different antipsychotic drugs.^6,18,19^ Our finding that the TAAR1 agonist ralmitaront reduces dopamine synthesis capacity suggests it could target this aspect of the pathophysiology of schizophrenia through a novel mechanism that potentially has fewer side effects than D_2/3_ blockers.^6,20^ These results also add to previous findings by the group that increased DSC caused by subchronic ketamine treatment can be decreased by TAAR1 agonism.^16^

The findings that acute cocaine did not have an effect on DSC in mice is interesting as it is one of the most widely used models to screen for antipsychotic drugs. The dose of cocaine used in the present study was used as a result of its validation in the earlier one.^14^ It is interesting to see that in spite of the fact that acute cocaine does not influence DSC in the present study, it has been reported that TAAR1 agonists and partial agonists reduce cocaine-induced elevated locomotor activity as well as dopamine overflow.^9,21^ Although k_loss_ mean values of groups were not statistically different from the control group, significant differences between the cocaine group and other treatment groups should be noted. The results seen with ralmitaront could be due to its ability to modulate dopamine transmission by interacting with TAAR1's functional interaction with dopamine transporters or influence ventral tegmental area dopamine neuron firing and hence further studies are needed to evaluate the impact of each of the above-mentioned mechanisms.^22^

Several limitations should be considered. One shortcoming of the study is that only a single dose of ralmitaront has been evaluated so we do not have a dose-response relationship, but our finding establishes a proof of principle. Future studies would be useful to test dose-response relationships. Similarly, a single exposure to cocaine has been evaluated in this study while a longer exposure could have different results. Another consideration is that we have used the modified Ki parameter of Holden et al,^15^ and it should be noted that it was developed for a setup where dynamic blood samples are drawn and not developed for use with the tissue reference method. However, this approach of graphical analysis expanded to include situations when only specific tissue regions are sampled and when trapping of the test substance is not completely irreversible has been derived, and though not directed specifically for FDOPA, it has been used for the current scenario.^23^ Also, direct comparison to an antipsychotic drug would have helped with comparisons.

## Conclusion

The TAAR1 partial agonist ralmitaront reduced presynaptic dopamine synthesis capacity when given either alone or in combination with cocaine indicating possible therapeutic use in disorders characterized by striatal hyperdopaminergia. There is an unmet need for new therapeutic agents to treat psychosis, especially agents that can lower presynaptic DSC without the side effects seen with current antipsychotics and TAAR1 agonists could play a role in that toolbox.

## Supplemental Material

sj-pdf-1-mix-10.1177_15353508241299546 - Supplemental material for Effect of the TAAR1 Partial Agonist Ralmitaront on Presynaptic Dopamine Synthesis Capacity Measured Using [^18^F]DOPA PET in Naïve and Cocaine-Treated Mice Supplemental material, sj-pdf-1-mix-10.1177_15353508241299546 for Effect of the TAAR1 Partial Agonist Ralmitaront on Presynaptic Dopamine Synthesis Capacity Measured Using [^18^F]DOPA PET in Naïve and Cocaine-Treated Mice by David R. Bonsall, PhD, Michelle Kokkinou, PhD, Els F. Halff, PhD, Grazia Rutigliano, PhD, Sac-Pham Tang, MSc, Mattia Veronese, PhD, Elaine E. Irvine, PhD, Dominic J. Withers, FRCP, PhD, Lisa A. Wells, PhD, Sridhar Natesan, PhD, Irene Gerlach, PhD, Štefan Holiga, PhD, Marius C. Hoener, PhD and Oliver D. Howes, MRCpsych, PhD in Molecular Imaging
