# Supplementary material for: Effect of the TAAR1 Partial Agonist Ralmitaront on Presynaptic Dopamine Synthesis Capacity Measured Using [18F]DOPA PET in Naïve and Cocaine-Treated Mice
Source: Mol Imaging. 2024 Dec 18;23:15353508241299546. doi: 10.1177/15353508241299546 (PMC11911367; doi:10.1177/15353508241299546)
Supplement: sj-pdf-1-mix-10.1177_15353508241299546 - Supplemental material for Effect of the TAAR1 Partial Agonist Ralmitaront on Presynaptic Dopamine Synthesis Capacity Measured Using [18F]DOPA PET in Naïve and Cocaine-Treated Mice [file sj-pdf-1-mix-10.1177_15353508241299546.pdf]

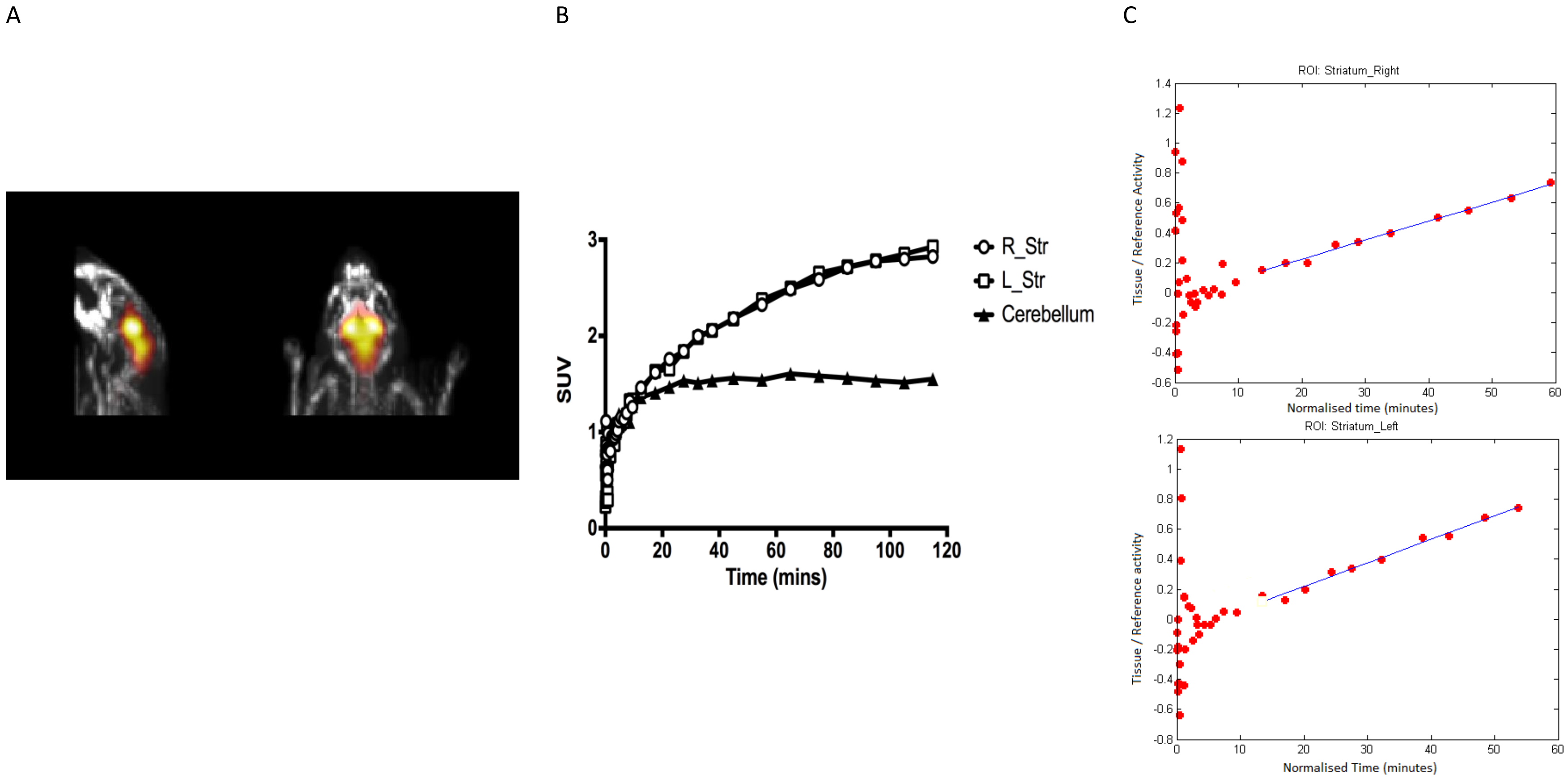


Figure 1.

A - Representative summated image of body weight-corrected, CT attenuated, [ 18$^{F}$]-DOPA striatal uptake of dynamic PET signal of a mouse from Group 1 (0 – 120 minutes). Regions of high uptake are shown in yellow and peak uptake is seen in the striatum.

B – Corresponding time activity curve from individual spherical ROIs drawn separately for the right and left striatum (0.06 $cm^3$) and a singular one for the cerebellum (0.1 $cm^3$). ROIs were placed within the skull as guided by CT and Franklin - Paxinos mouse atlas (2$^{nd}$ Edition, 2004, Academic Press, USA). Abbreviations: SUV - Standardized Uptake Value, Str – Striatum.

C – Corresponding operative Patlak plots of the right and left striatum.
